# Translation and Validation of the Gothenburg Trismus Questionnaire-2 into Italian Language

**DOI:** 10.3390/jcm14092949

**Published:** 2025-04-24

**Authors:** Andrea Frosolini, Lisa Tuomi, Olindo Massarelli, Caterina Finizia, Simone Benedetti, Lisa Catarzi, Andrea Lovato, Guido Gabriele, Paolo Gennaro

**Affiliations:** 1 Maxillofacial Surgery Unit, Department of Medical Biotechnologies, University of Siena, 53100 Siena, Italy; molindo74@gmail.com (O.M.); simone.benedetti1992@gmail.com (S.B.); lisa.catarzi@gmail.com (L.C.); guido.gabriele@unisi.it (G.G.); paolo.gennaro@unisi.it (P.G.); 2 Department of Otorhinolaryngology, Head and Neck Surgery, Sahlgrenska University Hospital, Region Västra Götaland, 41345 Gothenburg, Sweden; lisa.tuomi@gu.se (L.T.); caterina.finizia@orlss.gu.se (C.F.); 3 Department of Health and Rehabilitation, Institute of Neuroscience and Physiology, Sahlgrenska Academy, University of Gothenburg, 41345 Gothenburg, Sweden; 4 Department of Otorhinolaryngology, Head and Neck Surgery, Institute of Clinical Sciences, Sahlgrenska Academy, University of Gothenburg, 41345 Gothenburg, Sweden; 5 Otorhinolaryngology Unit, Department of Surgical Specialties, Vicenza Civil Hospital, 36100 Vicenza, Italy; andrea.lovato.3@hotmail.it

**Keywords:** trismus, head and neck cancer, temporomandibular disorders, Gothenburg Trismus Questionnaire, Italian

## Abstract

**Objectives:** This study aimed to translate and validate the Gothenburg Trismus Questionnaire-2 for Italian-speaking patients (I-GTQ2). **Methods:** A cross-sectional study was conducted with 200 participants. The translation process adhered to international standards. Patients completed the I-GTQ2 along with the European Organization for Research and Treatment of Cancer Quality of Life Questionnaire (EORTC QLQ) and the Hospital Anxiety and Depression Scale (HADS) to evaluate construct validity. Reliability was assessed using internal consistency and test–retest reliability (ICC). Known-group validity was also analyzed. **Results:** The I-GTQ2 showed high reliability, with Cronbach’s alpha ranging from 0.61 to 0.94 and ICC between 0.79 and 0.96. Known-group comparisons confirmed discriminative validity, with significant differences between patients with and without trismus in jaw-related problems (*p* = 0.005, *d* = 0.575) and large differences between patients and controls in most domains (*p* < 0.001, *d* > 0.65) except for muscular tension. Convergent validity was supported by strong correlations between GTQ-2 domains and EORTC QLQ-C30 (e.g., *r* = −0.54 for facial pain and global health status; *r* = 0.64 for jaw-related problems and pain) as well as moderate correlations with HADS anxiety (*r* = 0.39–0.52) and depression (*r* = 0.37–0.46). **Conclusions:** The I-GTQ2 is a reliable and valid tool for assessing the impact of trismus on the quality of life in Italian-speaking patients, and it is recommended for clinical and research use. Future studies should investigate its relationship with objective trismus measurements.

## 1. Introduction

Trismus, commonly referred to as restricted jaw movement or lockjaw, is a condition characterized by a limited range of motion of the jaw, affecting mouth opening. It can be temporary, typically resolving within two weeks, or permanent, significantly interfering with daily activities like speaking, eating, and swallowing. This condition complicates oral hygiene and dental procedures, profoundly impacting patients’ lives [1]. Beyond clinical symptoms, trismus may have significant socioeconomic consequences. Impairing oral intake and communication, it can interfere with employment and social interactions and lead to increased healthcare utilization due to the need for rehabilitation and nutritional support [1]. These limitations can substantially reduce patients’ quality of life and contribute to long-term disability and financial strain. Trismus arises from various etiologies, including acute causes like facial, mandibular, or iatrogenic trauma, and chronic causes such as temporomandibular disorders, neoplasia, and head and neck cancer treatments [2]. The prevalence of trismus is notably variable, often seen in patients undergoing radiation therapy for head and neck cancers, with a reported incidence of 38% to 42% in these cases [3]. Although trismus secondary to temporomandibular joint (TMJ) pathology is less common, temporomandibular disorders (TMD) are more prevalent—affecting up to 34% of the population, according to recent meta-analysis—thereby contributing to a significant global health burden [4]. Normal mouth opening ranges between 40 to 60 cm, with trismus generally defined as a maximum mouth opening of less than 36 mm. Diagnosing trismus is mainly clinical (by measuring the distance between the incisors) and supported by imaging techniques, mainly computed tomography and magnetic resonance imaging, to determine its etiology and involvement of the TMJ [5,6], while other tests such as electormyografy have been scarcely applied [7]. While trismus can often be self-limited, resolving with conservative treatments like heat therapy and NSAIDs, it may become chronic, especially in cases of fibrosis from radiotherapy, necessitating more intensive surgical and rehabilitative interventions [1,8].

Patient-reported outcome measures (PROMs) provide an essential patient perspective on the physical, functional, and psychological impacts of a disease and its treatment [9]. In the context of a symptom that potentially affects several vital functions, such as trismus, the evaluation of the patient’s quality of life is pivotal. Even though several different instruments have been applied, a specifically developed PROM is available for evaluating trismus: the Gothenburg Trismus Questionnaire [10]. Originally developed in 2012 (GTQ-1), the GTQ has undergone revisions to enhance its relevance and accuracy. The GTQ-2 [11], resulting from the inclusion of patient input and an expert panel review, now contains 29 items. These are divided into four domains: jaw-related problems (eight items), eating limitations (four items), muscular tension (three items), and a newly identified domain, facial pain (five items). The other questions consist of two single items capturing the impact of facial pain on the ability to participate in social, leisure, and family activities; one question regarding limitation on mouth opening; and six questions related to possible training of the ability to open the mouth. Furthermore, the GTQ-2 also includes a picture of a face, which allows the patient to localize the pain. Domains and single items range from 0 to 100, where 100 equals a high symptom burden, and 0 represents no symptoms.

The GTQ-2 shows high internal consistency (Cronbach’s alpha 0.74–0.96) across all domains, which is crucial for ensuring reliability in its measurements. Additionally, the questionnaire has excellent test–retest reliability (intraclass correlation coefficients ranging from 0.95 to 0.98). Construct validity was assessed by examining how well the questionnaire’s domains correlate with relevant domains of established quality of life measures, specifically the European Organization for Research and Treatment of Cancer Quality of Life Questionnaire Core 30 (EORTC QLQ-C30). The domains of GTQ-2, such as jaw-related problems and eating limitations, showed strong correlations with these established measures. This means that the GTQ-2’s assessment of trismus aligns well with how trismus affects quality of life aspects as measured by well-established tools. Moreover, the inclusion of patient input in the revision process has led to a questionnaire that is more aligned with patients’ experiences and perceptions, leading to the inclusion of a section focused on rehabilitation, ultimately enhancing its content validity and relevance. The GTQ-2 is available free of charge and can be used both in clinical settings for evaluating the impact of trismus on patients and in research [11]. To the best of our knowledge, the GTQ-2 stands as the most comprehensive, reliable, and valid instrument for assessing trismus. Furthermore, this instrument has been recently transculturally translated and validated in Indian, Chinese, and French populations [12,13,14].

In the context of modern healthcare and the growing integration of artificial intelligence (AI), the role of PROMs has become increasingly significant [15,16]. PROMs like the GTQ-2 help ensure that the patient’s perspective remains central, even as digital tools streamline clinical workflows and decision making. Their use contributes not only to individual care but also to the development of AI-driven systems that incorporate quality of life and symptom burden into personalized care planning. The digital evolution, including the use of electronic PROMs (ePROs), supports this integration and reinforces the importance of validated instruments for symptom monitoring in both routine care and research settings. In this evolving scenario, tools such as the GTQ-2 remain essential for human-centered, data-informed, clinical decision making.

Although precise national statistics regarding trismus prevalence in Italy are lacking, head and neck cancers represent a notable public health concern, with an estimated 9900 new cases diagnosed in 2020 (7300 in men and 2600 in women) and approximately 57,900 individuals living with a previous diagnosis of head and neck cancer [17]. Given the relatively high prevalence and functional burden of head and neck tumors in the Italian population, addressing trismus in this group is of both clinical and socioeconomic importance. Even though a range of treatments is available—from stretching exercises to surgical interventions in severe cases [8,9]—there remains a lack of patient-reported outcome measures (PROMs) in the Italian language for comprehensive evaluation. This gap underscores the need for translating and validating reliable instruments like the Gothenburg Trismus Questionnaire-2 into Italian. The primary objective of this study was to translate and validate the Gothenburg Trismus Questionnaire-2 into Italian. This will provide Italian-speaking clinicians and researchers a crucial tool for assessing trismus, aiding in better understanding its impact on patients’ quality of life and guiding appropriate interventions.

## 2. Materials and Methods

### 2.1. Approvals, Translation, and Cross-Cultural Adaptation

This cross-sectional study was conducted in accordance with the Ethical Committee for Clinical Research at the University Hospital of Siena (acceptance no. 5/2023). After receiving the consent of the authors, the GTQ-210 was translated from Swedish to Italian by two independent translators, according to international guidelines [18]. Following this, a consensus meeting involving an expert group, including members of our research team and a clinician experienced with the patient groups, was conducted to synthesize these translations into one Italian GTQ-2 version (referred to as I-GTQ2). The consensus version of I-GTQ2 was then re-translated into Swedish for comparison with the original version to ensure the translation’s accuracy. Upon satisfactory alignment with the original wording, the I-GTQ2 was approved for further use.

### 2.2. Pilot Study

The I-GTQ2 underwent a pilot study involving outpatients diagnosed with trismus due to temporomandibular disorders (TMD) or head and neck cancer (HNC) and who were capable of understanding and completing the I-GTQ2 questionnaire, additional validation questions, and semi-structured interview. The exclusion criteria included cognitive impairments and patients with trismus caused by conditions other than TMD or HNC, or those who did not provide informed consent. Ten participants answered the I-GTQ2 along with additional validation questions (see Supplementary Material S1). A semi-structured interview was then conducted with each participant to delve deeper into understanding the questionnaire items, particularly focusing on any difficulties encountered. The feedback gathered from these interviews did not result in any changes to the wording of the I-GTQ2. The questionnaire is available in Supplementary Material S2 and can be accessed for clinical or research purposes upon request to the corresponding author.

### 2.3. Validation Study

For the validation study, we included 100 participants with trismus and 100 age- and gender-matched healthy controls without trismus. Both groups were recruited through consecutive sampling from the outpatient clinics of the Maxillofacial Surgery Unit of the University Hospital of Siena during the study period. All individuals meeting the inclusion criteria were invited to participate. Eligible participants included patients diagnosed with TMD or HNC-related trismus, characterized by a maximal interincisal opening (MIO) of less than 36 mm [19]. For the control group, age- and gender-matched individuals were also recruited from the same hospitals. The control group consisted of patients who had no history of trismus but were treated for other common HNC-related conditions (e.g., oral cysts, impacted third molar, etc.) and had an MIO greater than 35 mm and no history of surgery in the preceding 3 months. Individuals of both groups had no cognitive impairments, were informed of the study’s aims and procedures, and agreed to participate by providing informed consent. To assess the reliability of the I-GTQ2, a test–retest procedure was implemented: thirty participants completed the I-GTQ2 a second time within two weeks of their first response. All data were collected between August 2023 and August 2024. All participants responded to a sociodemographic questionnaire alongside the I-GTQ2, including comorbidity that was assessed using the Adult Comorbidity Evaluation (ACE-27) and MIO, age, gender, marital status, smoking status, duration of trismus, and cancer location (for HNC) or reason for TMD. HNC and TMD participants also responded to the European Organization for Research and Treatment of Cancer Quality of Life Questionnaire Core Module (EORTC QLQ C30) [20] and Hospital Anxiety and Depression Scale (HADS) questionnaires [21]. The EORTC QLQ-C30 includes both functional and symptom scales. The functional scales cover domains such as physical functioning, role functioning, emotional functioning, cognitive functioning, and social functioning. Higher scores on these functional scales indicate better quality of life and functioning. Conversely, the symptom scales measure aspects such as fatigue, pain, nausea, and other disease-related symptoms, where higher scores denote greater symptom burden and poorer quality of life. In this study, we expected greater trismus severity to correlate negatively with the functional scales of the EORTC QLQ-C30, indicating reduced quality of life, and positively with its symptom scales, reflecting a higher symptom burden. The HADS consists of two subscales: one for anxiety (HADS-A) and one for depression (HADS-D), each containing seven items scored on a scale from 0 to 3. Higher scores on each subscale indicate greater levels of anxiety or depression. We expected positive correlations between trismus-related domains and the HADS anxiety (HADS-A) and depression (HADS-D) scores, suggesting that individuals with more severe trismus symptoms experience increased psychological distress.

### 2.4. Statistical Analysis

Descriptive statistics were calculated using standard methods. For categorical variables—all of which were nominal—the choice of statistical test depended on the structure of the contingency table and the distribution of expected cell frequencies. The chi-square test was used when comparing groups with tables equal to or larger than 2 × 2 and when all expected frequencies were ≥5. When expected frequencies in any cell were <5—particularly in 2 × 2 tables—Fisher’s exact test was applied to ensure statistical accuracy. For continuous variables, the Mann–Whitney U test was used, as the data did not follow a normal distribution. In addition to *p*-values, effect sizes (ES) were reported to quantify the magnitude of group differences: Cohen’s *d* for continuous variables and Cramer’s *V* or the phi coefficient for categorical variables, depending on the size of the contingency table. Effect sizes were interpreted using the thresholds proposed by Sullivan and Feinn [22]. For Cohen’s *d*, values of 0.2, 0.5, 0.8, and 1.3 were considered small, medium, large, and very large, respectively; for categorical variables, effect sizes were calculated using Cramer’s *V* or the phi (φ) coefficient, depending on the dimensionality of the contingency table. Interpretation followed Cohen’s guidelines, where values of 0.1, 0.3, and 0.5 were considered small, medium, and large, respectively. Correlations among the domains of the I-GTQ2, EORTC, and HADS were also assessed using Spearman correlation coefficients: correlation coefficients of 0.10–0.29 = weak association; 0.30–0.49 = moderate correlation; 0.50 or larger = strong correlation [23]. It was hypothesized that the correlations observed in the Italian validation study would resemble those found in the original Swedish validation study. Internal consistency was measured using Cronbach’s alpha coefficient. Test–retest reliability was determined by calculating the intraclass correlation coefficient (ICC) between scores from the first and second administration of the questionnaire. To evaluate known-group validity, the GTQ-2 domain scores were compared between patients in the study group and those in the control group. Floor and ceiling effects were defined as the proportions of patients scoring at the minimum or maximum levels, respectively. According to McHorney et al. (1995), the presence of floor or ceiling effects, indicated by more than 15% of patients scoring at the extremes, suggests that the response scale may lack adequate discrimination, and the items or domains may require re-evaluation [24]. The sample size of 200 subjects was determined based on findings from previous validation studies [11,12,13,14]. This specifically refers to the guidelines by Fayers and Machin, which recommend a minimum of five respondents per item [25]. Post hoc power analyses were conducted using Cohen’s *d* to estimate the observed power (1–β) for key group comparisons Statistical power was interpreted as adequate when the observed power (1–β) was ≥0.80 and low when <0.80. Statistical analysis was conducted using Jamovi 2.3 (The Jamovi Project 2022, Sidney, Australia), with a significance level set at *p* < 0.05 for all tests.

## 3. Results

### 3.1. Transcultural Adaptation and Pilot Study

Some linguistic challenges arose during the transcultural adaptation phase, such as translating “mouth opening wide” from Swedish (“gapa stort”). The initial term “completamente” (completely) was revised to “in modo ampio” (open wide) for accuracy. Similarly, “dispositivo per allungare la bocca” (mouth stretching device) was changed to “dispositivo per la divaricazione buccale” to enhance clarity for Italian speakers. These adjustments ensured linguistic accuracy and preserved the original meaning of the GTQ-2. Feedback from the pilot study confirmed that the translations were well received and understood. The final I-GTQ2 version is available in the additional materials.

### 3.2. Study Population

The study population consisted of 100 participants divided into two groups: 20 patients with HNC and 80 patients with TMD. These were compared to 100 age- and gender-matched healthy controls, as depicted in Table 1. The average age of the HNC and TMD patients was 47.3 years (SD = 20) and 43.2 years (SD = 14), respectively. The MIO showed a significant difference between HNC/TMD patients and controls, with the patient group having a mean MIO of 29.4 mm (SD = 7.5) compared to 44 mm (SD = 5.4) in the controls (*p* < 0.001; ES 0.238). All patients in the HNC group underwent surgical treatment (100%), with four patients (20%) also receiving radiotherapy. The average time since the completion of treatment was 31.3 months (SD 22.08, range 1–72). Among the 80 participants with TMD, the specific cause identified include disc displacement (n = 20), arthritis (n = 5), and muscular disorders (n = 29). For the remaining 26 participants, the cause of TMD was not recorded, and thus, this information is classified as missing data. The patient groups were compared to the healthy control group, which had a younger mean age and, consequently, a lower proportion of retired individuals and higher educational levels (Table 1).

### 3.3. Reliability

The internal consistency analysis, as measured by Cronbach’s alpha, revealed a strong reliability in most domains, as reported in Table 2. The jaw-related problems and facial pain subscales exhibited excellent internal consistency with alpha values of 0.94 and 0.94, respectively, indicating high reliability. The eating limitation subscale showed good internal consistency with an alpha of 0.84, while the muscular tension subscale had a lower alpha of 0.61, suggesting moderate internal consistency.

The test–retest reliability analysis involved 35 subjects over a two-week interval and demonstrated excellent average reliability, with ICCs ranging between 0.79 and 0.96 indicating moderate stability of responses between time points, as reported in Table 2.

### 3.4. Validity

**Figure 1:**
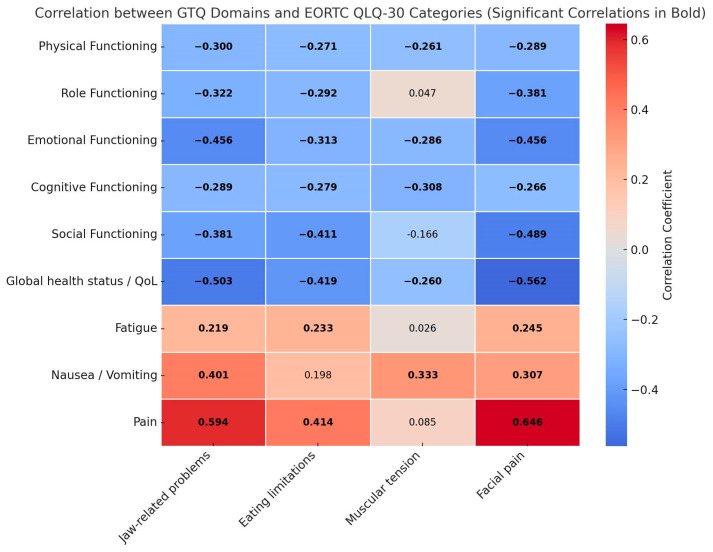
Spearman’s correlation between I-GTQ2 Domains and EORTC QLC-30 categories; statistical significance (*p* < 0.05) is depicted in bold. GTQ = Gothenburg Trismus Questionnaire, EORTC QLQ-C30: European Organisation for Research and Treatment of Cancer Quality of Life Questionnaire—Core 30.

Construct validity was evaluated by analyzing the correlations between the domains of the I-GTQ2 and the domains of the EORTC QLQ C30. Spearman correlation coefficients revealed several strong associations, particularly highlighting the alignment between trismus-related problems and the physical and functional impacts on patients’ quality of life. For the EORTC C30, the instrument demonstrated moderate to strong statistically significant correlations in the majority of cases (31 out of 36), as shown in Figure 1.

**Figure 2:**
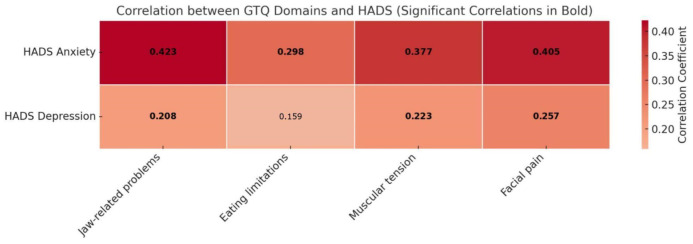
Spearman’s correlations between I-GTQ2 Domains and HADS; statistical significance (*p* < 0.05) is depicted in bold. GTQ = Gothenburg Trismus Questionnaire-2; HADS = Hospital Anxiety and Depression Scale.

Notable strong correlations included a negative association between jaw-related problems and facial pain with global health status/QOL (rho = −0.504, *p* < 0.001 and rho = −0.562, *p* < 0.001, respectively), as expected. Conversely, as anticipated, there were strong positive correlations between jaw-related problems and facial pain with the pain domain (rho = 0.594, *p* < 0.001 and rho = 0.646, *p* < 0.001, respectively). The construct validity of the I-GTQ2 was further assessed by analyzing its correlations with the HADS, as shown in Figure 2.

The analysis revealed several positive moderate associations between the GTQ-2 domains and both anxiety and depression (with rho values ranging from 0.208 to 0.423), and seven out of eight correlations were statistically significant (see Figure 2).

For the known-group validity analysis, the I-GTQ2 effectively distinguished between patients (merged TMD and HNC group) with and without trismus across multiple domains. As shown in Table 3, significant differences were observed between the trismus and non-trismus groups in terms of MIO, as expected.

Patients with trismus consistently reported higher scores than non-trismus groups for all I-GTQ2 domains except for muscular tension. Such scores were statistically significant for jaw-related problems (43.8 ± 23.3 vs. 20.8 ± 16.5, *p* < 0.01; ES 0.575). When comparing the trismus’ group patients with controls, higher scores were found across all domains, which was statistically significant for jaw-related problems (40.3 ± 24.2 vs. 7 ± 11.6, *p* < 0.01; ES 0.782), eating limitations (41.2 ± 20.6 vs. 14.3 ± 21.3, *p* < 0.01; ES 0.665), and facial pain (34.5 ± 25.6 vs. 5.6 ± 12.5, *p* < 0.01; ES 0.654). A post hoc power analysis revealed that all comparisons between trismus and non-trismus subgroups within the patient cohort were underpowered (1–β < 0.8), likely due to the small size of the non-trismus group. In contrast, the comparisons between patients and controls demonstrated adequate power (1–β > 0.99) for jaw-related problems, facial pain, and eating limitations. However, the comparison for muscular tension remained underpowered despite yielding a small-to-moderate effect size.

Table 4 provides descriptive statistics for the Italian version of the GTQ-2 in participants with trismus.

Across domains, the jaw-related problems and eating limitations showed moderate mean scores of 44.5 and 43.5, respectively, with minimal floor and ceiling effects. The muscular tension domain displayed a lower mean score (27.1) and a notable floor effect (26.4%), suggesting that a substantial portion of participants experienced minimal muscular tension. The facial pain domain had a mean score of 36.1, with 20.8% reporting no pain (floor effect), indicating variability in pain experiences among participants.

## 4. Discussion

This study aimed to perform a translation, cultural adaptation, and validation of the GTQ2 into Italian. The transcultural adaptation process highlighted several linguistic and cultural challenges that were addressed to preserve the questionnaire’s meaning and relevance. Feedback from pilot testing indicated that the revisions were well received, confirming that the adapted instrument maintained its core meaning while being more accessible to the target population. This process not only enhances the instrument’s usability but also reinforces the need for careful consideration of language and culture in health research [26].

The internal consistency analysis of the questionnaire revealed strong reliability across most domains, as indicated by Cronbach’s alpha values ranging from 0.61 to 0.94 (see Table 2). The muscular tension subscale exhibited the lower alpha, indicating only moderate internal consistency, as reported in previous translation studies [12,13,14]. For instance, the French validation study found a Cronbach’s alpha of 0.60 for the muscular tension domain, which aligns with our results [14]. Similarly, in the Chinese and Indian adaptation, low values of muscular tension subdomain were reported (0.75 and 0.54, respectively), supporting the reliability of the I-GTQ2 muscular tension measurements within this context [12,13]. Given that muscular tension consistently poses challenges in achieving high reliability across various populations and translations, this pattern merits further investigation. This may indicate that items within this domain are interpreted inconsistently by respondents or fail to comprehensively capture the complexities of muscular tension experienced by patients. It is also important to consider that this subscale comprises only three items, and Cronbach’s alpha is known to be sensitive to the number of items in a scale—often producing lower values in short subscales [27]. While a threshold of 0.70 is typically preferred, values between 0.60 and 0.70 are considered acceptable in exploratory studies or for subscales with few items [28]. Therefore, this domain should be approached with caution, and it may be beneficial to supplement it with objective measures of muscular tension in future research [29,30]. All previous versions of the GTQ-2 noted strong internal consistency across the other three domains [12,14]. The Cronbach’s alpha values in this study were generally high, reflecting excellent reliability for the instrument as a whole, reaffirming its effectiveness as a tool for assessing trismus-related quality of life. In the test–retest phase, ICC findings supported the reliability of the questionnaire, although individual variability should be noted. This may reflect fluctuations in the intensity or perception of symptoms—particularly in domains such as facial pain and muscular tension—which can vary over short periods due to stress, daily activities, or individual pain thresholds [31]. Such variability is expected in self-reported outcomes and does not necessarily undermine the instrument’s reliability at the group level, but it should be considered when interpreting changes at the individual level.

The strong correlations between the GTQ-2 domains and several key quality of life domains from the EORTC QLQ-C30 confirm the construct validity of the I-GTQ2, demonstrating its relevance and sensitivity in capturing the impact of trismus on various aspects of patients’ lives. For the EORTC QLQ-C30, the instrument performed as expected, showing several moderate to strong correlations similar to the original validation study [11]. The moderate correlations between GTQ-2 domains and both HADS anxiety and total scores indicate that trismus significantly affects the emotional well-being of patients, particularly in terms of anxiety, as expected [32]. The associations between facial pain, muscular tension, and jaw-related problems with anxiety and overall emotional distress further validate the use of the I-GTQ2 for assessing the psychological impact of trismus.

The known-group comparisons confirmed the I-GTQ2’s validity in differentiating between patients with and without trismus, particularly regarding jaw-related problems. The absence of statistical significance in other domains, such as eating limitations and facial pain, is likely due to the small subgroup sample sizes. When the sample was expanded by comparing patients to controls, significant differences emerged in three out of the four domains, with muscular tension being close to significance but not reaching it. The lack of significance for muscular tension may be linked to its lower reliability and to higher values in our control group compared to other studies [14]. Post hoc power analysis revealed that all comparisons within the trismus and non-trismus subgroups were underpowered (1–β < 0.80), reflecting the small sample size in the non-trismus subgroup (n = 8–9) and limiting the strength of conclusions drawn from these comparisons. In the analysis comparing HNC/TMD patients with controls, statistical power was adequate (1–β > 0.99) for all domains except the muscular tension (1–β = 0.114). These findings highlight the importance of interpreting non-significant results within the context of sample size limitations and emphasize the recruitment of larger, adequately powered cohorts in future studies targeting subgroup comparisons.

The translation and validation of the GTQ-2 into Italian demonstrated several strengths. One major strength was the comprehensive adaptation process, which ensured that the Italian version of the questionnaire retained linguistic and cultural relevance while preserving the core meaning and structure of the original GTQ-2 without losing its clinical focus. Another strength lay in the inclusion of both pilot and validation studies with adequate design and population, which assure the statistical power of the findings and support the generalizability of the results to the Italian trismus patients cohorts. Despite these strengths, the study also revealed some limitations. The medical treatment for the included patient from test to retest need to be taken into account since ongoing therapy was not an exclusion criteria of the study. Although the overall sample size was adequate, subgroup analyses were underpowered, especially for the HNC population, leading to non-significant findings despite clinical relevance. Another limitation of the study was the heterogeneity of trismus etiology within the TMD group, as inclusion was based solely on restricted mouth opening without distinguishing between muscular and articular mechanisms (MRI could have helped clarify the underlying cause, and future research may benefit from correlating GTQ-2 results with imaging findings). Lastly, the study’s generalizability was somewhat limited by its single-center setting. Expanding the validation to more diverse hospitals and clinics and more diverse patient groups with other causes of trismus would have enhanced the utility of the I-GTQ2 across a broader spectrum of trismus patients.

The availability of a validated Italian version of the GTQ-2 offers several practical benefits. Clinically, it provides healthcare professionals with a standardized and patient-centered tool to assess the severity and impact of trismus, supporting better symptom monitoring and individualized care planning. In research settings, it enables cross-cultural comparisons and inclusion of Italian-speaking populations in multicenter studies involving PROMs. Additionally, its digital adaptability makes it suitable for integration into electronic health records and AI-enhanced decision support systems, reinforcing the role of PROMs in modern, data-informed healthcare.

## 5. Conclusions

The I-GTQ2 was successfully translated and demonstrated to be both reliable and valid. It effectively discriminated jaw-related problems between patients with and without trismus in this single-center population study. We recommend the use of this instrument for both clinical and research purposes. Future studies should consider exploring its correlation with objective measures for further validation.

## Figures and Tables

**Table 1:** Sociodemographic and clinical characteristics of the study participants and controls. *p*-values are from Mann–Whitney U test for continuous variables, Fisher’s exact test, or chi-square for categorical variables. Effect sizes: Cohen’s *d* for continuous variables, Cramer’s *V* or phi for categorical variables.

	Study Group		*p*-value (Effect Size)	Study Group HNC + TMD (n = 100)	Controls (n = 100)	*p*-Value (Effect Size)
	HNC (n = 20)	TMD (n = 80)				
	Mean (SD)			Mean (SD)	Mean (SD)	
Age (years)	64.3 (14.1)	42.7 (18.9)	<0.001 (0.0762)	47.3 (20)	43.2 (14)	0.360 (0.238)
MIO (mm)	31 (6.8)	29 (7.7)	0.336 (0.9064)	29.4 (7.5)	44 (5.4)	<0.001 (2.234)
	n (%)			n (%)		
Gender						
Male	11 (55)	43 (53.7)	0.815 (0.08)	54 (54)	46 (46)	0.258 (0.070)
Female	9 (46)	36 (45)		45 (45)	54 (27)	
Non binary	0 (NA)	1(1.25)		1 (1)	0 (NA)	
Marital status						
Single	4 (20)	35 (43.7)	0.219 (0.043)	39 (39)	43 (43)	0.658 (0.032)
Married/Cohabitant	12 (80)	42(52.5)		54 (54)	50 (50)	
Education level						
Elementary school	7 (35)	17 (21.2)	0.210 (0.292)	27 (27)	10 (10)	<0.001 (0.292)
High school	12 (60)	47 (58.7)		59 (59)	52 (52)	
University	1 (5)	15 (18.7)		16 (16)	38 (38)	
Occupational status						
Working	6 (30)	54 (67)	<0.001 (0.399)	60 (60)	58 (58)	<0.001 (0.399)
Retired	12 (60)	14 (17)		26 (26)	4 (4)	
Studying	1 (5)	9 (11.2)		10 (10)	36 (36)	
Smoking						
Never smoked	9 (46)	51 (63.7)	0.177 (0.071)	60 (60)	48 (48)	0.588 (0.071)
Stopped smoking	6 (30)	11 (12.5)		27 (27))	26 (26)	
Current smoker	5 (25)	18 (22.5)		23 (23)	26 (26)	
Tumor location						
Oral cavity	15 (75)	NA	NA			
Nasopharynx	1 (5)	NA				
Oropharynx	4 (20)	NA				
Cause of TMD						
Discal	NA	20 (25)	NA			
Arthritis	NA	5 (6.25)				
Muscular	NA	29 (36.25)				

HNC = head and neck cancer; TMD = temporomandibular disorders; MIO = maximum interincisal opening; NA = Not Aplicable; SD = standard deviation.

**Table 2:** Reliability estimates.

I-GTQ2 Domains	Internal Consistency ^a^	Test–Retest ^b^
		ICC (CI)
Jaw-related problems	0.94	0.96 (0.94–0.973)
Eating limitation	0.84	0.89 (0.84–0.93)
Muscular tension	0.61	0.79 (0.68–0.87)
Facial pain	0.94	0.95 (0.93–0.97)

^a^ Cronbach’s α. ^b^ Test–retest sample size n = 35. CI = confidence interval; I-GTQ2 = Italian version of the Gothenburg Trismus Questionnaire-2; ICC = intraclass correlation coefficient.

**Table 3:** Mean I-GTQ2 scores and statistical significance comparing patient subgroups (with and without trismus) as well as a broader comparison between all patients and controls. *p*-values were calculated using the Mann–Whitney U test, and effect sizes are reported as Cohen’s *d*.

	HNC/TMD Non-Trismus Mean (SD)	HNC/TMD Trismus Mean (SD)	*p*-Value (Effect Size)	Post Hoc Analysis	HNC/TMD Mean (SD)	Controls Mean (SD)	*p*-Value (Effect Size)	Post Hoc Analysis
Age (years)	54.9 (25.2)	47.1 (19.6)	0.445 (0.167)	0.073	47.3 (20)	43.2 (14)	0.360 (0.076)	0.082
MIO (mm)	52.3 (4.1)	27.8 (6.20)	<0.001 (1.0)	0.756	29.4 (7.5)	44 (5.4)	<0.001 (0.906)	1.0
Jaw-related problems	20.8 (16.5)	43.8 (23.3)	0.005 (0.575)	0.363	40.3 (24.2)	7 (11.6)	<0.001 (0.782)	1.0
Eating limitations	31.3 (17.7)	42.9 (20.7)	0.111 (0.344)	0.15	41.2 (20.6)	14.3 (21.3)	<0.001 (0.665)	0.997
Muscular tension	26.9 (21.2)	26.9 (23.7)	0.834 (0.044)	0.052	25.2 (23.4)	19.0 (16.6)	0.196 (0.104)	0.114
Facial pain	22.8 (20.2)	36.8 (24.7)	0.131 (0.310)	0.14	34.5 (25.6)	5.6 (12.5)	<0.001 (0.654)	0.995

TMD = temporomandibular disorders; HNC = head and neck cancer; MIO = maximum interincisal opening; SD = standard deviation.

**Table 4:** Descriptives of the I-GTQ2 for the participants with trismus.

Domains	Items (n)	Range	Mean	Median	SD	Floor n (%)	Ceiling n (%)
Jaw-related problems	8	0–93.7	44.5	46.9	23	11 (11)	5 (5.5)
Eating limitation	4	0–87.5	43.5	40.6	20.2	5 (5.5)	1 (1.4)
Muscular tension	3	0–83.3	27.1	25	23.6	26 (26.4)	0 (0)
Facial pain	5	0–87.5	36.1	35	24.2	20 (20.8)	1 (1.4)

n = number; SD = standard deviation.

## Data Availability

The raw data supporting the conclusions of this article will be made available by the authors on request.

## Supplementary Materials

The supporting information (additional validation question and Italian GTQ-2) can be downloaded at https://www.mdpi.com/article/10.3390/jcm14092949/s1.

## Abbreviation List

GTQ-2	Gothenburg Trismus Questionnaire-2
I-GTQ2	Italian version of the Gothenburg Trismus Questionnaire-2
PROM	Patient-Reported Outcome Measure
TMD	Temporomandibular Disorders
HNC	Head and Neck Cancer
TMJ	Temporomandibular Joint
MIO	Maximum Interincisal Opening
EORTC QLQ-C30	European Organisation for Research and Treatment of Cancer Quality of Life Questionnaire—Core 30
HADS	Hospital Anxiety and Depression Scale
ICC	Intraclass Correlation Coefficient
SD	Standard Deviation
ES	Effect Size
CI	Confidence Interval
ePRO	Electronic Patient-Reported Outcome
AI	Artificial Intelligence
NA	Not Applicable
QOL	Quality of Life
