# Supplementary material for: Translation and Validation of the Gothenburg Trismus Questionnaire-2 into Italian Language
Source: J Clin Med. 2025 Apr 24;14(9):2949. doi: 10.3390/jcm14092949 (PMC12073072; doi:10.3390/jcm14092949)
Supplement: Supplementary file 1 [file jcm-14-02949-s001.zip › jcm-3577368-supplementary.pdf]

Questionario I-GTQ2 (**Italian - Göteborg Trismus Questionnaire version 2)**

Supplementary materials for the manuscript by Frosolini et al., published in 2025 in the *Journal of Clinical Medicine*. For usage or reproduction, please contact the corresponding author at andreafrosolini@gmail.com.

Questo questionario contiene domande relative al trisma (difficoltà di apertura della bocca) ed ai problemi a livello mascellare-mandibolare. Rispondi alle domande selezionando la risposta che ritieni più appropriata. In caso di incertezza, indica l'opzione che ti sembra più appropriata.

Durante **l'ultima settimana** hai avuto:

		Per nulla<br>1	Un po'<br>2	Moderatamente<br>3	Molto<br>4	Moltissimo<br>5
1.	Affaticamento a livello mascellare	☐	☐	☐	☐	☐
2.	Rigidità a livello mascellare	☐	☐	☐	☐	☐
3.	Dolore facciale	☐	☐	☐	☐	☐
4.	Dolore a livello mascellare	☐	☐	☐	☐	☐
5.	Dolore durante i movimenti mascellari (sbadiglio/masticazione)	☐	☐	☐	☐	☐
6.	Difficoltà ad aprire la bocca in modo ampio	☐	☐	☐	☐	☐
7.	Dolore alla muscolatura mascellare	☐	☐	☐	☐	☐
8.	Difficoltà a sbadigliare	☐	☐	☐	☐	☐
9.	Rumori all'articolazione temporomandibolare	☐	☐	☐	☐	☐

In che misura ritieni che le seguenti attività siano limitate dai problemi a livello mascellare-mandibolare?

		Per nulla<br>1	Un po'<br>2	Moderatamente<br>3	Molto<br>4	Moltissimo<br>5
10.	Mangiare cibi duri	☐	☐	☐	☐	☐
11.	Inserire il cibo in bocca	☐	☐	☐	☐	☐
12.	Mangiare cibi morbidi	☐	☐	☐	☐	☐
13.	Mordere	☐	☐	☐	☐	☐

Ti succede di:

		Per nulla<br>1	Un po'<br>2	Moderatamente<br>3	Molto<br>4	Moltissimo<br>5
14.	Stringere con forza i denti	☐	☐	☐	☐	☐
15.	Spingere con la lingua	☐	☐	☐	☐	☐

Nelle domande 16-20 si parla di dolore facciale e ci si riferisci a dolori facciali e/o mandibolari in relazione alla capacitá di apertura della bocca

		Non ho avuto dolore facciale	Leggero	Moderato	Severo	Molto severo
16.	Quale è stata l'intensità massima del tuo dolore facciale **nelle ultime 24 ore**?	☐ 1	☐ 2	☐ 3	☐ 4	☐ 5
17.	Quale è stata l'intensità massima del tuo dolore facciale **nell'ultima settimana**?	☐ 1	☐ 2	☐ 3	☐ 4	☐ 5
18.	Quale è stata l'intensità, mediamente, del tuo dolore facciale **nell'ultima settimana**?	☐ 1	☐ 2	☐ 3	☐ 4	☐ 5

		Non ho avuto dolore facciale	Leggero	Moderato	Severo	Molto severo
19.	Il dolore facciale ha influenzato la tua capacità di partecipare ad attività sociali, ricreative e familiari?	☐ 1	☐ 2	☐ 3	☐ 4	☐ 5
20.	Il dolore facciale ha influenzato la tua capacità di lavorare (incluso sia il lavoro retribuito che i lavori domestici)?	☐ 1	☐ 2	☐ 3	☐ 4	☐ 5

		Per nulla	Un po'	Moderatamente	Molto	Moltissimo
21.	Quanto è stata limitata, **nelle ultime 24 ore**, la tua capacità di aprire la bocca?	☐ 1	☐ 2	☐ 3	☐ 4	☐ 5

		Non ho avuto una capacità di apertura limitata	Un po'	Moderatamente	Molto	Moltissimo
22.	La tua limitazione nell' aprire la bocca ha influenzato la tua capacità di partecipare ad attività sociali, ricreative e familiari?	☐ 1	☐ 2	☐ 3	☐ 4	☐ 5
23.	La tua limitazione nell' aprire la bocca ha influenzato la tua capacità di lavorare (incluso sia il lavoro retribuito che i lavori domestici)?	☐ 1	☐ 2	☐ 3	☐ 4	☐ 5

Indica le parti del viso in cui senti dolore. Puoi scegliere più di un'opzione. Se non hai alcun dolore facciale, salta questa domanda.

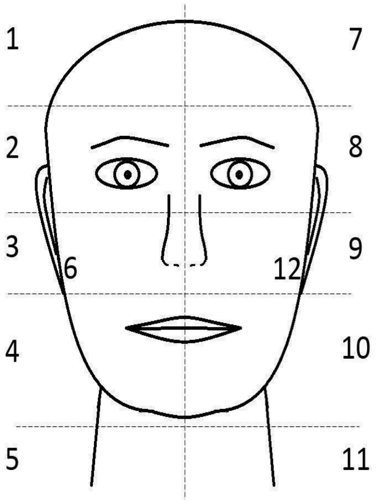

Lato destro del viso		Lato sinistro del viso	
1. Fronte destra	☐	7. Fronte sinistra	☐
2. Attorno all'occhio destro	☐	8. Attorno all'occhio sinistro	☐
3. Il centro del volto destro	☐	9. Il centro del volto sinistro	☐
4. Bocca/mento destro	☐	10. Bocca/mento sinistro	☐
5. Collo destro	☐	11. Collo sinistro	☐
6. Articolazione temporomandibolare destra	☐	12. Articolazione temporomandibolare sinistra	☐

24. Ti alleni per migliorare la tua capacità di aprire la bocca? (dispositivo per la divaricazione della bocca, stretching con le dita, altro)

☐ Sì ☐ No Se si, come ti alleni?____

Rispondi alle domande da 25 a 29 solo se ti alleni per migliorare la tua capacità di apertura della bocca

	Per nulla<br>1	Un po'<br>2	Moderatamente<br>3	Molto<br>4	Moltissimo<br>5
25. Trovi scomodo utilizzare la tua attrezzatura di esercitazione/allenarti per migliorare la tua capacità di aprire la bocca?	☐	☐	☐	☐	☐
26. Trovi fisicamente faticoso utilizzare la tua attrezzatura di esercitazione/allenarti per migliorare la tua capacità di aprire la bocca?	☐	☐	☐	☐	☐
27. Trovi che gli esercizi che ti sono stati consigliati richiedano molto tempo?	☐	☐	☐	☐	☐
28. Pensi che gli esercizi siano efficaci, ti aiutano?	☐	☐	☐	☐	☐

29. Quante volte al giorno in media ti alleni?

Mai	Una volta al giorno	Due volte al giorno	Tre volte al giorno	Quattro volte al giorno	Cinque o più volte al giorno
☐ 0	☐ 1	☐ 2	☐ 3	☐ 4	☐ 5
